# Risk perception of coronavirus disease 2019 (COVID-19) and its related factors among college students in China during quarantine

**DOI:** 10.1371/journal.pone.0237626

**Published:** 2020-08-13

**Authors:** Yani Ding, Xueying Du, Qinmei Li, Miao Zhang, Qingjun Zhang, Xiaodong Tan, Qing Liu

**Affiliations:** 1 Department of Epidemiology and Health Statistics, School of Health Sciences, Wuhan University, Wuhan, China; 2 Hubei Center for Disease Control and Prevention, Wuhan, Hubei, China; National University of Singapore, SINGAPORE

## Abstract

**Objective:**

At the end of 2019, the outbreak of coronavirus disease 2019 (COVID-19) in Wuhan was a serious threat to public health. This study aimed to evaluate the risk perception of COVID-19 among college students in China during the quarantine, explore its related factors, and provide reference for future study.

**Methods:**

This study invited college students from various provinces of China to participate in the survey through the Internet, and a total of 1,461 college students were included. T-test and analysis of variance were used to explore the relationship between demographic characteristics, social pressure, knowledge and risk perception. Multiple linear regression was used to identify factors associated with risk perception.

**Results:**

This study shows that college students in China have high risk perception of COVID-19. Female college students (p<0.01), non-medical students (p<0.01), college students whose schools are located in Hubei (p = 0.01) and college students with higher knowledge level (p<0.01) have higher risk perception.

**Conclusion:**

Due to the strong infectivity and occult nature of COVID-19, it is necessary to improve the risk perception of college students through health education in various ways, and attention should be paid to some college students with low risk perception.

## Introduction

Since the end of 2019, coronavirus disease 2019 (COVID-19) was identified and broke out in Wuhan. It is one of the most recent emerging infectious diseases that can pose a serious threat to human health, cause great economic losses and cause panic. Common signs of COVID-19 infection include respiratory symptoms, fever, cough, wheezing and dyspnea. In more severe cases, the infection can lead to pneumonia, severe acute respiratory syndrome, kidney failure and even death [1, 2]. In order to prevent the epidemic from spreading out of control, Wuhan announced the lockdown of the city on January 23, and the country began to implement control, with the government urging people to stay at home and limit to go out.

At present, the mortality rate of COVID-19 is lower than SARS (severe acute respiratory syndrome) and Middle East respiratory syndrome (MERS) [3]. However, the global transmission of COVID-19 is rapid because of the direct human-to-human infection, which makes it challenging to adequately inform the public about the risks involved and precautions needed. Clearly, the true risk from COVID-19 virus might be low, but this epidemic has received broad media attention and been subjected to social media discussion, which may have induced the perception of risk among people, which in turn might determine their behaviors [4, 5]. Therefore, understanding their risk perception could be an effective method to communicate the health policy. Previous study on infectious diseases has also shown the impact of population movements on the rapid spread of epidemics and the advantages of strict health and quarantine measures in preventing epidemics [6]. In the case of a pandemic, public health authorities will depend on the willingness and ability of the public to comply with recommendations regarding personal hygiene, vaccination or prevention, quarantine, travel restrictions or closure of public buildings such as schools [7]. Compliance with recommended preventive actions is not self-evident. The control of outbreaks of infectious diseases depends on the cooperation of the entire population and the need to increase protection for individuals [8]. A factor that may affect willingness and motivation to take preventive actions is risk perception [4, 5, 9]. Risk perception belongs to the category of psychology, which refers to an individual's perception and understanding of various objective dangers in the outside world. Risk perception is an important factor influencing risk behaviors. People with lower risk perception tend to take risk behaviors or reduce preventive behaviors [10], while people with high risk perception tend to take preventive behavior [11]. In addition, people do perceive various aspects of risk perceptions differently for different emerging infectious diseases. The risk perception for emerging infectious diseases such as SARS and avian influenza were amongst the highest rated in the present study, especially in case of an outbreak. From a public health perspective, this offers a good starting point for risk communication and precautionary actions [12]. Risk perception is affected by a variety of factors [13]. Although risk perception has been well studied in some areas, such as environmental risk [14, 15], there is no study on college students' risk perception of COVID-19 in China.

College students, as one of the most dynamic groups in China, have great mobility, strong mobility and like to socialize. They are young, healthy, and often have mild symptoms after being infected with COVID-19, which can have a significant impact on the spread of COVID-19. Besides, the outbreak of COVID-19 coincided with the time of massive transportation before the Spring Festival. College students on winter vacation, as an important part of the massive transportation, may have played a role in the spread of COVID-19 to the whole country. College students also has a great impact on the risk perception of the people around them. Therefore, it is necessary to pay particular attention to the risk perception of COVID-19 among college students in China. In order to evaluate the risk perception of COVID-19 among college students in China and explore its related factors in this period and provide further evidence for prevention and control of COVID-19. We conducted a questionnaire survey on the risk perception of COVID-19 among college students in different provinces of China in February 2020, when the COVID-19 epidemic was in the outbreak and college students were mostly isolated at home. By the end of March 2020, although the epidemic of COVID-19 in China has been basically controlled, the epidemic has also spread globally and has become a global pandemic [16], causing closure of many schools. Therefore, we believe this study also has great significance for epidemic control in other countries.

## Materials and methods

### Participants and procedures

The purpose of this survey was to explore the risk perception of COVID-19 among college students in China and its related factors. The respondents were college students in China (including junior college student, Undergraduate, postgraduate and doctoral student). We posted the questionnaire on WeChat and QQ (social software commonly used in China), and invited college students from different regions and majors to complete the questionnaire voluntarily. Meanwhile, we also sent the questionnaire to the university teachers who had cooperated with us, and used their contact network to spread the questionnaire. Before the investigation, we stated the purpose of the investigation, and we will continue the investigation after obtaining the consent. This questionnaire is anonymous and can only be filled in once for one IP address. The survey began on February 4, 2020, and ended on February 7, 2020, when China was in the period of outbreak of COVID-19. During this period, emergency control measures have been implemented in all Chinese cities, including schools closed, public gatherings banned, entertainment places closed and traffic restricted. Therefore, most people had to stay at home. Incomplete questionnaires cannot be submitted. We excluded illogical answers (for example, age is not realistic). In the end, a total of 1,461 valid questionnaires were collected.

### Questionnaire

This questionnaire (S1 and S2 Files) is designed by our team members, reviewed and revised by epidemiology experts before investigation to establish the content validity. Our data is made up of four parts. The first part is the demographic characteristics including gender, age, grade, major, school location and home location (6 items). The second part is about social pressure, including whether family member or friends have been diagnosed with COVID-19, whether family member or friends have been exposed to confirmed or suspected patient of COVID-19, and the physical condition of the parents(3 items). The third part is the knowledge, with a total of 7 items. These items evaluated the knowledge related to the incubation period, vulnerable groups, symptoms, transmission routes, preventive measures, correct hand washing and mask wearing, with a total score of 20 points. The fourth part is risk perception, which is evaluated on a 5-point scale (totally disagree, disagree, hard to say, agree, strongly agree). There are 4 items with a total score of 20. The four items are: Even if a person is in good health, he may be infected by COVID-19; I was more likely to be infected by COVID-19 than anyone else; Someone once reminded me to be careful of COVID-19; I would worry about my family getting infected with COVID-19. The reliability of our questionnaire evaluated using the Cronbach alpha was 0.64. The data of our study can be accessed from supplement (S3 File).

### Ethics statement

The approval was obtained from The Institution Review Board at Wuhan University (IRB#:2020YF0026). Our investigation is anonymous, and identifiable personal information was not collected. Every participant was informed about and understood the purpose of our investigation before entering the study.

### Statistical analysis

Our study was analyzed using SAS 9.0. Mean and standard deviation are used to describe the risk perception score. T test and variance analysis were used to evaluate the relationship between demographic characteristics, social pressure, knowledge and risk perception. In order to explore the relationship between risk perception and various variables, we use the multiple linear regression, and established the three models. The gender, grade, major and school location are included in the first model; the social pressures are included in the second model on the basis of the first model. The third layer adds knowledge as the independent variable on the basis of the second layer. The first group of each variable was selected as a reference. All tests’ statistical significances were defined as bilateral P<0.05.

## Results

### General characteristics

According to Table 1, a total of 1,461 college students were investigated in this study, of which 639 (43.7%) were males and 822 (56.2%) were females. Mean for age in our study is 20.7. Junior college students and undergraduate students account for 95.1% (1,390), postgraduate students and doctoral students account for 95.1% (1,390). Medical majors account for the highest proportion, accounting for 4.9% (71), while other majors account for 56.7% (828). There are 710 (48.6%) participants of schools in Wuhan, 67 (4.6%) in other areas in Hubei province except Wuhan and 684 (46.8%) in other provinces in China except Hubei province. There are 75 (5.1%) participants whose home are located in Wuhan, 191 (13.1%) in other areas in Hubei province except Wuhan and 1,195 (81.8%) in other provinces in China except Hubei province. 52 (3.6%) participants, their family members or friends have been diagnosed with COVID-19. 74 (5.1%) participants, their family members or friends have been exposed to confirmed or suspected patients of COVID-19. 1,450 (99.2%) participants’ parents were in good health, and 11 (0.8%) were in poor health.

**Table 1:** Distribution of general characteristics of college students in China (n = 1,461).

Characteristics	Number	%
**Gender**		
Male	639	43.7
Female	822	56.3
**Grade**		
Junior college student and Undergraduate	1,390	95.1
Postgraduate and doctoral student	71	4.9
**Major**		
Medicine	633	43.3
Non-medicine	828	56.7
**School location**		
Wuhan	710	48.6
Other areas in Hubei province except Wuhan	67	4.6
Other provinces in China except Hubei province	684	46.8
**Home location**		
Wuhan	75	5.1
Other areas in Hubei province except Wuhan	191	13.1
Other provinces in China except Hubei province	1,195	81.8
**Whether you, your family members or friends have been diagnosed with COVID-19**		
Yes	52	3.6
No	1,409	96.4
**Whether you, your family members or friends have been exposed to confirmed or suspected patients of COVID-19**		
Yes	74	5.1
No	1,387	94.9
**The physical condition of the parents**		
Good	1,450	99.2
Poor	11	0.8

COVID-19, coronavirus disease 2019.

### Risk perception of COVID-19

Among 4 items of risk perception, 92.5% (1,351) of college students believe that even if a person in good health could be infected by COVID-19, 6.4% (94) of college students thought they were more likely to be infected by COVID-19, 80.4%(1,175) of college students said they were reminded to be careful of COVID-19, and 85.1% (1,244) of college students would worry about my family getting infected with COVID-19. The mean risk perception score of all participants is 14.965, and the standard deviation is 2.003. These results show that college students have a high risk perception of COVID-19.

Table 2 shows the risk perception scores and their results of univariate analysis among college students in China with different characteristics. Gender and whether they have been exposed to confirmed or suspected cases of COVID-19 were the influencing factors of risk perception. Female had a higher level of risk perception than male (p<0.01). The risk perception of college students whose family member or friends have been exposed to confirmed or suspected patient of COVID-19, was higher than that of college students who had not been exposed (p<0.01).

**Table 2:** Univariate analysis of risk perception among college students in China with different socio-demographic factors.

Characteristics	Risk perception score	T value	P value
	Mean ± SD		
**Gender**		-3.2	<0.01
Male	14.8±2.19		
Female	15.1±1.8		
**Grade**		-1.1	0.26
Junior college student and Undergraduate	15.0±2.0		
Postgraduate and doctoral student	15.2±1.9		
**Major**		1.4	0.16
Medicine	14.9±2.1		
Non-medicine	15.0±1.8		
**School location**		1.2 ^a^	0.30
Wuhan	15.0±2.0		
Other areas in Hubei province except Wuhan	15.2±2.0		
Other provinces in China except Hubei province	14.9±2.1		
**Home location**		0.6^a^	0.57
Wuhan	15.1±2.2		
Other areas in Hubei province except Wuhan	15.1±2.0		
Other provinces in China except Hubei province	14.9±2.0		
**Whether you, your family members or friends have been diagnosed with COVID-19**		-1.8	0.08
Yes	15.6±2.4		
No	14.9±2.0		
**Whether you, your family members or friends have been exposed to confirmed or suspected patients of COVID-19**		-2.5	0.01
Yes	15.5±2.2		
No	14.9±2.0		
**The physical condition of the parents**		-1.4	0.16
Good	15.0±2.0		
Poor	15.8±1.7		

^a^ F value of variance analysis.

SD, standard deviation; COVID-19, coronavirus disease 2019.

Table 3 shows the risk perception scores and their results of univariate analysis among college students in China with different knowledge of COVID-19. For all participants, only 25.5% of them thought that droplets and contact transmission were the transmission route of COVID-19, 50.7% of participants believed that the population was generally susceptible to COVID-19, 82.8% of participants knew about the incubation period of COVID-19, 66.5% of participants knew the common symptoms of COVID-19, 47.4% of participants were unaware of the correct way to wash their hands, 33.1% of participants were unaware of the correct way to wear masks, and 30.8% of participants were unaware of the measures to prevent COVID-19. Among seven items of COVID-19 knowledge, knowing susceptible population (p<0.01), symptoms of infection (p = 0.02), proper hand washing (p = 0.03), proper wearing of mask (p<0.01), preventive measures (p<0.01) were associated with risk perception of COVID-19, and the group that answered correctly had a higher risk perception than those who answered incorrectly.

**Table 3:** Univariate analysis of risk perception among college students in China with different knowledge of COVID-19.

Knowledge of COVID-19	Knowing knowledge of COVID-19		Not knowing knowledge of COVID-19		T value	P value
	Number (%)	Risk perception score	Number (%)	Risk perception score		
		Mean ± SD		Mean ± SD		
Transmission route	372 (25.5)	15.0±0.1	1,089 (74.5)	15.0±0.1	<0.1	0.97
Susceptible population	741 (50.7)	15.2±1.8	720 (49.3)	14.7±2.1	4.8	<0.01
Incubation period	1,210 (82.8)	15.0±1.9	251 (17.2)	14.7±2.3	1.7	0.09
Symptoms of infection	972 (66.5)	15.1±2.0	489 (33.5)	14.8±2.0	2.3	0.02
Proper hand washing	768 (52.6)	15.1±2.0	693 (47.4)	14.8±2.0	2.1	0.03
Proper wearing of mask	977 (66.9)	15.1±1.9	484 (33.1)	14.7±2.1	3.4	<0.01
Preventive measures	1,011 (69.2)	15.1±1.9	450 (30.8)	14.7±2.3	3.2	<0.01

SD, standard deviation; COVID-19, coronavirus disease 2019.

The results of multivariable linear regression of risk perception among college students in China are shown in Table 4. Gender was significant in model 1 and model 2 (P <0.01). According to the results of model 3, gender, Major, school location, and knowledge level are highly correlated with risk perception. Gender was associated with risk perception of COVID-19 (P <0.01), and female college students have a higher risk perception of COVID-19. Major had association with risk perception of COVID-19 (P <0.01). The risk perception of medical students is lower than that of non-medical students. school location was associated with their risk perception of COVID-19 (P <0.05). Students studying in other parts of Hubei province except Wuhan have a higher risk perception of COVID-19. Compared with Hubei province, the risk perception of college students studying outside the province is relatively low. There was a significant positive correlation between knowledge level and risk perception (P <0.01), that is, the higher the knowledge level of college students, the higher their risk perception of COVID-19.

**Table 4:** Multivariable linear regression of risk perception among college students in China.

	Model 1		Model 2		Model 3	
	B value	P value	B value	P value	B value	P value
**Gender**	0.40	<0.1	0.39	<0.1	0.28	<0.01
**Grade**	0.32	0.19	0.26	0.28	0.25	0.30
**Major**	0.21	0.14	0.21	0.14	0.41	<0.01
**School location (Other provinces in China except Hubei province is the reference)**						
Wuhan	0.06	0.67	0.05	0.75	0.04	0.78
Other areas in Hubei province except Wuhan	0.39	0.16	0.40	0.15	0.62	0.01
**Home location (Other provinces in China except Hubei province is the reference)**						
Wuhan	0.02	0.94	-0.13	0.62	-0.11	0.67
Other areas in Hubei province except Wuhan	0.07	0.69	0.03	0.87	0.02	0.91
**Whether you, your family members or friends have been diagnosed with COVID-19**			0.38	0.22	0.40	0.20
**Whether you, your family members or friends have been exposed to confirmed or suspected patients of COVID-19**			0.48	0.06	0.48	0.05
**The physical condition of the parents**			0.76	0.21	0.65	0.27
**Knowledge of COVID-19**					0.16	<0.01
R^2^	0.01		0.02		0.05	
Adjusted R^2^	0.01		0.01		0.04	
F value	2.79		2.84		6.34	
P value of the model	<0.01		<0.01		<0.01	

In model 1, gender, grade, major, school location and home location were included; in model 2, whether you, your family members or friends have been diagnosed with COVID-19, whether you, your family members or friends have been exposed to confirmed or suspected patients of COVID-19 and the physical condition of the parents were added; in model 3, knowledge of COVID-19 was added.

COVID-19, coronavirus disease 2019.

## Discussion

COVID-19 has become a social problem of global concern [16]. There is no specific treatment, and the treatment is mainly symptomatic [2, 17]. Although the outbreak in China has been basically under control, the outbreak in the rest of the world still cannot be underestimated. The control of any infectious disease requires the continuous efforts of the medical community and the whole society [18, 19]. It is an effective way to prevent COVID-19 infection and control the spread of COVID-19 to carry out the public health publicity centered on COVID-19 in order to appropriately improve the risk perception of the public, and finally make them take the initiative to take preventive measures. There are some studies on risk perception of COVID-19 in other countries. Nearly half of the participants in a study of India felt panic by reports of COVID-19 [20]. A study in Iran showed that medical students had a medium risk perception of COVID-19 [21]. In a study in Italy, most participants felt uncertainty, fear, and sadness [22]. However, there are still no studies on risk perception of Chinese college students. As a high-knowledge group, college students are not only the mainstay of the future national construction, but also can spread their knowledge and strong risk awareness to the people around them [23]. Therefore, it is particularly important to understand the risk perception level of college students on COVID-19 and its related factors. The participants of our survey are college students from various provinces, covering a wide range of areas, which to a large extent reflects the risk perception of COVID-19 among college students in China.

The results show that most college students have a higher risk perception and a certain knowledge of COVID-19. Although most college students were aware of the risk of COVID-19, 7.5% disagreed with the statement that a person in good health could be infected by COVID-19. Underestimation of this risk characteristic may lead to risk behaviors and neglect of early symptoms of COVID-19. Considering the strong infectivity of COVID-19 and the occult nature of the disease [24, 25], a small number of low-risk perceived population's risk behaviors may lead to recurrent outbreaks. Most college students said that they were warned about COVID-19, which to some extent reflects the public's attention to COVID-19.85.1% of college students were concerned that their family members getting infected with COVID-19, only a minority thought they were more likely to be infected by COVID-19. This may be because it is the winter vacation now. Most college students stay at home, and their families may come into contact with people from all over the country because of their work or taking care of the family, so college students believe their parents have higher risk of COVID-19 and their own risk is lower. Another possible reason is that older people are more likely to worse prognosis after infection with COVID-19. So college students may be more worried about their parents and other elderly family members than themselves. The third possible reason is that college students lack the subjective felling of control over their parents' behavior. The measures reducing the feeling of control may increase their risk perception [26]. Parents' behavior is out of their control and they worry about risky behavior from their families. Thus, college students have higher risk perception of their parents than that of themselves. Our study shows that college students in China have a high risk perception of COVID-19, which is conducive to the epidemic prevention work of the society [27]. However, there are still a small number of college students whose risk perception is not high enough, which should attract our attention. At present, the epidemic prevention work in China is at a critical moment, and the perception on the risk of COVID-19 cannot be reduced. In the future, it is necessary to strengthen the publicity work on the risk of COVID-19 to further improve the risk perception of COVID-19 among all college students.

The results of univariate analysis (Tables 3 and 4) in this study showed that female had a higher level of risk perception than male, which is consistent with the results of our multivariable linear regression. The risk perception of college students whose family member or friends have been exposed to confirmed or suspected patient of COVID-19, was higher than that of college students who had not been exposed. The possible reason is awareness of the strong infectivity and close contact with COVID-19, which leads to the increase in the risk perception of COVID-19. Among the 7 items of knowledge, 5 items are significant. In these 5 items, 49.3% of college students don't know that people are generally susceptible to COVID-19. It may be that people generally thought that the elderly were more susceptible than the young in the early stage of the epidemic, which misled the young to some extent, resulting in the lower risk perception. About a third of college students don't know the symptoms and preventive measures of covid-19. Although the government and health authorities propagandized symptoms of COVID-19 and proposed preventive measures, not everyone would pay attention to them. The importance attached to this information partly reflects the greater attention paid to the epidemic. People with comprehensive knowledge are more aware of the risk and have a higher risk perception. This suggests that knowledge is related to risk perception of COVID-19. Although the college students have given some attention, but the knowledge of COVID-19 is not comprehensive enough. In addition, the study of Huynh (2020) indicated that risk perception has high correlation to the misuse of facial masks [28]. Therefore, it is very important to wear masks correctly [29].

The multivariable linear regression showed that there was a statistically significant difference between the risk perception scores of college students of different genders. Female college students have a higher risk perception of COVID-19, which is consistent with a study of SARS in Holland [11] and a study of MERS in South Korea [13]. The reason may be that female are sensitive, their thoughts are more delicate, and their sensitivity to the risk of COVID-19 is higher. Major is also related to risk perception of COVID-19. Compared with non-medical students, medical students have lower risk perception. This result is similar to the study of college students' attitudes toward H1N1 in Turkey [30]. A study of college students' psychological response to SARS in Hong Kong reported that healthcare students had lower perceived stress than non-healthcare students [31]. This may be due to the medical expertise of medical students making them have better understanding of COVID-19 than non-medical students. Non-medical students lacking medical expertise may be excessively worried about the damage caused by COVID-19, result in a higher risk perception and even feel anxious and panic. In addition, our study was conducted during the winter vacation. Most medical students were at home on vacation, did not participate in clinical practice, and would not being directly exposed to diagnosed or suspected cases of COVID-19. This also explains why medical students' risk perception of COVID-19 is not higher than that of non-medical students. The school location of the college students had association with their risk perception of COVID-19, and students studying in Hubei province have a higher risk perception. It may be related to the fact that Hubei province is both the outbreak site and the epicenter of the epidemic, and the university students studying in Hubei province are more or less in contact with crowded places such as railway stations and shopping malls. Compared with Hubei province, the epidemic was not so serious when college students returned home. Therefore, the risk perception of college students studying in the latter region is lower than that of students studying in Hubei province. In addition, students returning home from Hubei province (including Wuhan and non-Wuhan cities) will be required to self-quarantine for 14 days, which will undoubtedly increase their risk perception. But the situation outside the province is relatively easy, the epidemic situation is well controlled, the university students studying outside the province have relatively limited contact with the epidemic area and suspected cases. At that time, Hubei province implemented a total blockade, but the provincial management is relatively lax. Therefore, college students outside the province have a lower perception of COVID-19 risk than college students in the province. In addition, the higher the knowledge level, the higher the risk perception of COVID-19. This finding is consistent with some previous studies of MERS, which show that knowledge is positively correlated with risk perception [32–34]. The higher the knowledge level of COVID-19, the more knowledge they have about its transmission mode, main symptoms and preventive measures, and the more they can fully realize that COVID-19 has a strong infectious power, a long incubation period, improve risk perception, and the disease is hidden and difficult to identify[24, 25], so the risk perception level is higher.

The spread of the outbreak also coincided with the peak travel time during the Spring Festival. As one of the most mobile groups in China, college students have acted as a very important viral carrier in this outbreak, which accelerated the spread of the epidemic. After that, the peak of college students returning to school is about to come. Therefore, while strengthening the management of college students returning to school, it is particularly important for college students to fully understand and perceive the risk of COVID-19. At present, although most college students generally have a high knowledge level of COVID-19, there are still a small number of college students with a low knowledge level, which should be paid attention to by us. In addition, the college students as a higher education group, have higher knowledge level, they are good at following and learning the knowledge of COVID-19 through various channels, and their risk perception of outbreak of COVID-19 will affect people's risk perception around them, thus to the epidemic prevention behavior of social members and public health security. But the general public has limited access to COVID-19, especially in the elderly (the average age of patients with COVID-19 is now 51). Therefore, in addition to the promotion of COVID-19 knowledge by TV, Internet and other media, government departments at all levels and health institutions should be fully mobilized to cooperate closely, so that people can learn about this disease in a short period of time, pay attention to this disease, and take the initiative to prevent it on the basis of cognition. In addition, the study has shown that the overwhelming amount of information and the overuse of mass media in communicating the COVID-19 virus might contribute to overreaction, unwarranted public fear, and an overly pessimistic feeling in perceiving the current risk [35]. So when it comes to communicating information, the attitude of spreading information can shift from emergency response to preventive preparedness, and this would be likely to reduce people's fear and panic.

Our study has several limitations. First, due to the influence of the epidemic situation, Web-survey was used. There may be difficult to calculate response rates and a lack of representativeness of our study. However, we did not offer any reward for the participants who completed the questionnaire, which can reduce repeat responses [36]. Although the number of medical students in our study is relatively large, there is little change of risk perception after a weighted analysis of the major (the mean value of risk perception before and after weighted analysis are 14.97 and 15.02), which suggests that our study has some representativeness. And we can collect relevant information more reliably due to the anonymity of web-survey [37]. In addition, web-survey has good objectivity, can be free from space-time and geographical restrictions, fast, and a supplement to the traditional survey methods. It can help us to collect objective data on the risk perception of COVID-19 among college students in different provinces of China during quarantine. Second, there may be recall bias as our study used a self-administered questionnaire. Third, our study was cross-sectional design rather than longitudinal, which may lead us to be unable to estimate changes in risk perception over time, making the relationship between variables and risk perception more tentative. In addition, our study restricted to Chinese college students, so it can not reflect Chinese population of other age groups. However, our study shows the risk perception of COVID-19 among college students in China during quarantine and explores its influencing factors. It can provide a reference for long-term study in the future and provide a theoretical basis for government departments and health agencies to carry out effective prevention of COVID-19.

## Conclusions

Our study shows that college students in China have a high risk perception. However, there were differences among different genders, majors, school locations and knowledge levels. Female students, non-medical students, students whose schools are located in Wuhan and students with high knowledge level have higher risk perception. In view of the mobility and influence of college students in China, we should improve the knowledge level of some college students, so as to improve the risk perception, and let college students lead people around to change their attitudes.

## Supporting information

S1 File Original study questionnaire. (DOCX)

S2 File English study questionnaire. (DOCX)

S3 File Original data set. (XLS)

## References

[1] Chen N, Zhou M, Dong X, Qu J, Gong F, Han Y, et al Epidemiological and clinical characteristics of 99 cases of 2019 novel coronavirus pneumonia in Wuhan, China: a descriptive study. Lancet (London, England). 2020;395(10223):507–13. Epub 2020/02/03. 10.1016/s0140-6736(20)30211-7 . 32007143 PMC7135076

[2] Lake MA. What we know so far: COVID-19 current clinical knowledge and research. Clinical medicine (London, England). 2020;20(2):124–7. Epub 2020/03/07. 10.7861/clinmed.2019-coron . 32139372 PMC7081812

[3] Bassetti M, Vena A, Giacobbe DR. The novel Chinese coronavirus (2019-nCoV) infections: Challenges for fighting the storm. European Journal of Clinical Investigation. 2020;50(3):e13209 10.1111/eci.13209 32003000 PMC7163647

[4] Sjöberg L. Factors in Risk Perception. Risk Analysis. 2000;20(1):1–12. 10.1111/0272-4332.00001 10795334

[5] Weinstein ND. The precaution adoption process. Health psychology. 1988;7(4):355 10.1037//0278-6133.7.4.355 3049068

[6] Bell DM. Public health interventions and SARS spread, 2003. Emerg Infect Dis. 2004;10(11):1900 10.3201/eid1011.040729 15550198 PMC3329045

[7] World Health Organization Writing G, Bell D, Nicoll A, Fukuda K, Horby, Monto A, et al Non-pharmaceutical interventions for pandemic influenza, national and community measures. Emerg Infect Dis. 2006;12(1):88–94. 10.3201/eid1201.051371 . 16494723 PMC3291415

[8] Yan QL, Tang SY, Xiao YN. Impact of individual behaviour change on the spread of emerging infectious diseases. Statistics in medicine. 2018;37(6):948–69. Epub 2017/12/02. 10.1002/sim.7548 . 29193194

[9] Brewer NT, Chapman GB, Gibbons FX, Gerrard M, McCaul KD, Weinstein ND. Meta-analysis of the relationship between risk perception and health behavior: The example of vaccination. Health Psychology. 2007;26(2):136–45. 10.1037/0278-6133.26.2.136 17385964

[10] Adefuye AS, Abiona TC, Balogun JA, Lukobo-Durrell M. HIV sexual risk behaviors and perception of risk among college students: implications for planning interventions. BMC public health. 2009;9:281 Epub 2009/08/06. 10.1186/1471-2458-9-281 . 19653901 PMC2733304

[11] Brug J, Aro AR, Oenema A, de Zwart O, Richardus JH, Bishop GD. SARS risk perception, knowledge, precautions, and information sources, the Netherlands. Emerg Infect Dis. 2004;10(8):1486–9. 10.3201/eid1008.040283 . 15496256 PMC3320399

[12] de Zwart O, Veldhuijzen IK, Elam G, Aro AR, Abraham T, Bishop GD, et al Perceived threat, risk perception, and efficacy beliefs related to SARS and other (emerging) infectious diseases: results of an international survey. Int J Behav Med. 2009;16(1):30–40. Epub 01/06. 10.1007/s12529-008-9008-2 . 19125335 PMC2691522

[13] Yang S, Cho SI. Middle East respiratory syndrome risk perception among students at a university in South Korea, 2015. American journal of infection control. 2017;45(6):e53–e60. Epub 2017/04/08. 10.1016/j.ajic.2017.02.013 . 28385465 PMC7115287

[14] Gao S, Li W, Ling S, Dou X, Liu X. An Empirical Study on the Influence Path of Environmental Risk Perception on Behavioral Responses In China. International journal of environmental research and public health. 2019;16(16). Epub 2019/08/14. 10.3390/ijerph16162856 . 31405088 PMC6719179

[15] Grasmuck D, Scholz RW. Risk perception of heavy metal soil contamination by high-exposed and low-exposed inhabitants: the role of knowledge and emotional concerns. Risk analysis: an official publication of the Society for Risk Analysis. 2005;25(3):611–22. Epub 2005/07/19. 10.1111/j.1539-6924.2005.00628.x . 16022694

[16] Organization WH. Coronavirus disease (COVID-19) outbreak. URL https://www.who.int/emergencies/diseases/novel-coronavirus-2019. 2020.

[17] Adhikari SP, Meng S, Wu YJ, Mao YP, Ye RX, Wang QZ, et al Epidemiology, causes, clinical manifestation and diagnosis, prevention and control of coronavirus disease (COVID-19) during the early outbreak period: a scoping review. Infectious diseases of poverty. 2020;9(1):29 Epub 2020/03/19. 10.1186/s40249-020-00646-x . 32183901 PMC7079521

[18] Schantz PM, Tsang VCW. The US Centers for Disease Control and Prevention (CDC) and research and control of cysticercosis. Acta Tropica. 2003;87(1):161–3. 10.1016/s0001-706x(03)00039-1 12781391

[19] Zhang S, Liu N. The Evolutions of Medical Building Network Structure for Emerging Infectious Disease Protection and Control. Cell Biochemistry and Biophysics. 2014;70(3):1741–8. 10.1007/s12013-014-0123-1 25096502

[20] Roy D, Tripathy S, Kar SK, Sharma N, Verma SK, Kaushal V. Study of knowledge, attitude, anxiety & perceived mental healthcare need in Indian population during COVID-19 pandemic. Asian J Psychiatr. 2020;51:102083-. 10.1016/j.ajp.2020.102083 . 32283510 PMC7139237

[21] Taghrir MH, Borazjani R, Shiraly R. COVID-19 and Iranian Medical Students; A Survey on Their Related-Knowledge, Preventive Behaviors and Risk Perception. Archives of Iranian medicine. 2020;23(4):249–54. Epub 2020/04/10. 10.34172/aim.2020.06 . 32271598

[22] Motta Zanin G, Gentile E, Parisi A, Spasiano D. A Preliminary Evaluation of the Public Risk Perception Related to the COVID-19 Health Emergency in Italy. International journal of environmental research and public health. 2020;17(9):3024 10.3390/ijerph17093024 . 32349253 PMC7246845

[23] C S H, K M M, Handi P, Khavasi P, Doddmani SS, Riyas M. To Study the Level of Awareness About Complications of Chronic Suppurative Otitis Media (CSOM) in CSOM Patients. J Clin Diagn Res. 2014;8(2):59–61. Epub 02/03. 10.7860/JCDR/2014/8009.4008 . 24701483 PMC3972599

[24] Liu Y, Gayle AA, Wilder-Smith A, Rocklov J. The reproductive number of COVID-19 is higher compared to SARS coronavirus. Journal of travel medicine. 2020;27(2). Epub 2020/02/14. 10.1093/jtm/taaa021 . 32052846 PMC7074654

[25] Wang Y, Wang Y, Chen Y, Qin Q. Unique epidemiological and clinical features of the emerging 2019 novel coronavirus pneumonia (COVID-19) implicate special control measures. Journal of medical virology. 2020 Epub 2020/03/07. 10.1002/jmv.25748 . 32134116 PMC7228347

[26] Van Damme W, Van Lerberghe W. Epidemics and fear. Tropical medicine & international health: TM & IH. 2000;5(8):511–4. Epub 2000/09/20. 10.1046/j.1365-3156.2000.00599.x . 10995091

[27] Brewer NT, Weinstein ND, Cuite CL, Herrington JE. Risk perceptions and their relation to risk behavior. Annals of behavioral medicine: a publication of the Society of Behavioral Medicine. 2004;27(2):125–30. Epub 2004/03/18. 10.1207/s15324796abm2702_7 . 15026296

[28] Huynh TD. The more I fear about COVID-19, the more I wear medical masks: A survey on risk perception and medical masks uses. medRxiv. 2020:2020.03.26.20044388. 10.1101/2020.03.26.20044388

[29] Huynh TLD. “If You Wear a Mask, Then You Must Know How to Use It and Dispose of It Properly!”: A Survey Study in Vietnam. Review of Behavioral Economics. 2020;7(2):145–58.

[30] Akan H, Gurol Y, Izbirak G, Ozdatli S, Yilmaz G, Vitrinel A, et al Knowledge and attitudes of university students toward pandemic influenza: a cross-sectional study from Turkey. BMC public health. 2010;10:413-. 10.1186/1471-2458-10-413 . 20626872 PMC2918554

[31] Wong JGWS, Cheung EPT, Cheung V, Cheung C, Chan MTY, Chua SE, et al Psychological responses to the SARS outbreak in healthcare students in Hong Kong. Medical Teacher. 2004;26(7):657–9. 10.1080/01421590400006572 15763860

[32] Kim JS, Choi JS. Middle East respiratory syndrome-related knowledge, preventive behaviours and risk perception among nursing students during outbreak. Journal of clinical nursing. 2016;25(17–18):2542–9. Epub 2016/06/09. 10.1111/jocn.13295 . 27273475 PMC7166634

[33] Gautret P, Benkouiten S, Salaheddine I, Belhouchat K, Drali T, Parola P, et al Hajj pilgrims knowledge about Middle East respiratory syndrome coronavirus, August to September 2013. Euro surveillance: bulletin Europeen sur les maladies transmissibles = European communicable disease bulletin. 2013;18(41):20604 Epub 2013/10/19. 10.2807/1560-7917.es2013.18.41.20604 . 24135123

[34] Khan MU, Shah S, Ahmad A, Fatokun O. Knowledge and attitude of healthcare workers about Middle East Respiratory Syndrome in multispecialty hospitals of Qassim, Saudi Arabia. BMC public health. 2014;14:1281 Epub 2014/12/17. 10.1186/1471-2458-14-1281 . 25510239 PMC4300996

[35] Huynh TL. The COVID-19 risk perception: A survey on socioeconomics and media attention. Econ Bull. 2020;40(1):758–64.

[36] Bowen AM, Daniel CM, Williams ML, Baird GL. Identifying multiple submissions in Internet research: preserving data integrity. AIDS Behav. 2008;12(6):964–73. Epub 02/01. 10.1007/s10461-007-9352-2 . 18240015 PMC2615134

[37] van Gelder MMHJ, Bretveld RW, Roeleveld N. Web-based Questionnaires: The Future in Epidemiology? American journal of epidemiology. 2010;172(11):1292–8. 10.1093/aje/kwq291 20880962

