# Supplementary material for: Risk perception of coronavirus disease 2019 (COVID-19) and its related factors among college students in China during quarantine
Source: PLoS One. 2020 Aug 13;15(8):e0237626. doi: 10.1371/journal.pone.0237626 (PMC7425914; doi:10.1371/journal.pone.0237626)
Supplement: S1 File — (DOCX) [file pone.0237626.s001.docx]

**大学生对新型冠状病毒感染的肺炎认知-态度调查**

        大学生们，您好！为了解您对新型冠状病毒感染的肺炎相关知识和态度，武汉大学健康学院开展了本次网络调查。该调查以匿名的形式进行，绝对不会泄露您的任何个人信息。调查结果对今后疫情防控和健康教育意义重大，请花几分钟的时间参与并转发。谢谢！

**第一部分 基本信息**

1. 您的性别： [单选题] *

| ○男 | ○女 |  |  |  |  |  |  |
| --- | --- | --- | --- | --- | --- | --- | --- |

2. 您的年龄  (岁)： [填空题] *

_________________________________

3. 您就读学校位于: [填空题] *

_________________________________

4. 目前，您所在的城市为: [填空题] *

_________________________________

5. 您的年级 [单选题] *

| ○A.大一 | ○B.大二 | ○C.大三 | ○D.大四 | ○E.大五 |
| --- | --- | --- | --- | --- |
| ○F.研一 | ○G.研二 | ○H.研三 | ○I.博一 | ○J.博二 |
| ○K.博三 |  |  |  |  |

6. 您的专业 [单选题] *

| ○A.文科类 | ○B.理科类 | ○C.工科类 | ○D.农学类 | ○E.医学类 |
| --- | --- | --- | --- | --- |
| ○F.艺术类 |  |  |  |  |

**第二部分 社会压力**

7. 截至目前为止，本人及亲朋好友是否被确诊感染新型冠状病毒? [多选题] *

| □A.本人 | □B.家人或亲戚 | □C.朋友 |
| --- | --- | --- |
| □D.认识的其他人 | □E.否 |  |

8. 截至目前为止，本人及亲朋好友是否接触过感染新型冠状病毒的人或疑似病人？ [多选题] *

| □A.本人 | □B.家人或亲戚 | □C.朋友 |
| --- | --- | --- |
| □D.认识的其他人 | □E.否 |  |

9. 您父母的总体健康状况 [单选题] *

| ○A.健康 |
| --- |
| ○B.良好 |
| ○C.较差 |
| ○D.很差 |

**第三部分 认知**
以下问题是为了了解您对新型冠状病毒感染的肺炎的认识程度，请根据您的自身情况选择合适答案。此部分共20分。

10. 据您了解新型冠状病毒的传播途径有哪些 [多选题] * （1分）

正确答案为AB，本题完全答对得一分

| □A. 飞沫传播 | □B. 接触传播 | □C. 蚊虫传播 |
| --- | --- | --- |
| □D. 粪口传播 | □E. 不知道 |  |

11. 据您了解新型冠状病毒的易感人群是 [单选题] * （1分）

正确答案为E，本题完全答对得一分

| ○A.儿童易感 | ○B.青壮年人群易感 | ○C.中年人群易感 |
| --- | --- | --- |
| ○D.老年人群易感 | ○E.人群普遍易感 | ○F.不知道 |

12. 据您了解新型冠状病毒感染常见的症状有哪些？ [多选题] *（1分）

正确答案为ABCDE，本题完全答对得一分

| □A. 干咳 | □B. 发热 | □C. 呼吸急促、甚至困难 |
| --- | --- | --- |
| □D. 乏力 | □E. 精神差、食欲差 | □F. 不知道 |

13. 您认为以下哪些是预防新型冠状病毒感染的有效措施？ [多选题] *（9分）

正确答案为ABCDEHI，本题每选择一个正确选项得1分，没有选择错误选项得1分

答对一个算一分

| □A. 尽量少出门，不参加聚会 | □B. 避免接触武汉返乡人员及发热人员 |
| --- | --- |
| □C. 勤用洗手液、肥皂等洗手 | □D. 勤开窗通风 |
| □E. 外出时佩戴口罩 | □F. 服用病毒灵、达菲等 |
| □G. 熏醋 | □H. 使用医用酒精对衣物、随身物品等进行消毒 |
| □I. 打喷嚏或咳嗽时用纸巾或手肘捂住口鼻 |  |

14. 您是否了解正确洗手的方法？ [单选题] *（3分）

| ○A. 1=完全不了解 | ○B. 2=了解一点 | ○C.3= 一般了解 |
| --- | --- | --- |
| ○D. 4=了解很多 | ○E. 5=非常了解 |  |

15. 您是否了解正确佩戴口罩的方法？ [单选题] *（3分）

| ○A. 1=完全不了解 | ○B. 2=了解一点 | ○C. 3=一般了解 |
| --- | --- | --- |
| ○D. 4=了解很多 | ○E. 5=非常了解 |  |

16. 您认为以下关于新型冠状病毒感染的肺炎的说法正确的是？ [多选题] *

正确答案为AB，本题每选择一个正确选项得1分

| □A. 潜伏期一般为3-7天，最长不超过14天 |
| --- |
| □B. 在潜伏期，新型冠状病毒也具有传染性  □C. 不知道 |

**第四部分 风险感知**

以下问题是为了了解您对新型冠状病毒感染的肺炎的态度，答案没有对错，请根据您的自身感受，选择最适当的答案。

**1=完全不可能 2=不可能 3=不好说 4=可能 5=非常可能** 此部分共20分

17. 即使一个人身体好，也可能受到新型冠状病毒感染。 [单选题] *

| ○A. 完全不可能 | ○B. 不可能 | ○C. 不好说 |
| --- | --- | --- |
| ○D. 可能 | ○E. 非常可能 |  |

18. 与其他人相比,我更容易出现新型冠状病毒感染的肺炎。 [单选题] *

| ○A. 完全不会 | ○B. 不会出现 | ○C. 不好说 |
| --- | --- | --- |
| ○D. 会出现 | ○E. 肯定会 |  |

19. 有人曾提醒过我小心感染新型冠状病毒。 [单选题] *

| ○A. 从没人提醒 |
| --- |
| ○B. 偶尔提醒 |
| ○C. 不好说 |
| ○D. 经常有人提醒 |
| ○E. 时刻有人提醒 |

20. 我会担心我的家人受到新型冠状病毒的感染。 [单选题] *

| ○A. 完全不担心 | ○B. 不担心 | ○C. 不好说 |
| --- | --- | --- |
| ○D. 有些担心 | ○E. 非常担心 |  |

**再次感谢您的支持！**
