# Supplementary material for: Risk perception of coronavirus disease 2019 (COVID-19) and its related factors among college students in China during quarantine
Source: PLoS One. 2020 Aug 13;15(8):e0237626. doi: 10.1371/journal.pone.0237626 (PMC7425914; doi:10.1371/journal.pone.0237626)
Supplement: S2 File — (DOCX) [file pone.0237626.s002.docx]

**A cognition-attitude survey on COVID-19 among college students**

Hello, college students! In order to understand your knowledge and attitude about COVID-19, School of Health Sciences, Wuhan University launched this online survey. The survey was conducted anonymously and will never reveal any of your personal information. Please take a few minutes to participate and forward the findings, which are of great significance for epidemic prevention and health education in the future. Thank you very much!

**Part one Basic Information**

1. Your gender: [single choice] *

□1=Male □2=female

2. Your age [fill in the blanks]*

_________________________________

3. Your school is located in: [fill in the blank] *

_________________________________

4. Your home is located in: [fill in the blank] *

_________________________________

5. Your grade [single choice] *

□A. freshman □B. sophomore □C. junior □D. senior □E. senior □F. Grind one

□G. Grind two □H. Grind three □I. PhD one □J. PhD two □K. PhD three

6. Your major [single choice] *

□A. liberal arts □B. science □C. engineering □D. agriculture

□E. medicine □F. art

**Part two Social Pressure**

7. Have I and my family and friends been diagnosed with COVID-19 so far? [multiple choice] *

□A. myself □B. family or relatives □C. friends

□D. other people you know □E. no

8. Have I or my family or friends come into contact with a person or a suspected patient infected with COVID-19 so far? [multiple choice] *

□A. myself □B. family or relatives □C. friends

□D. other people you know □E. no

9. General health of your parents [single choice] *

□A. health

□B. good

□C. poor

□D. poorer

**Part three Knowledge of COVID-19**

The following questions are based on your knowledge of COVID-19. Please choose an appropriate answer according to your situation. (The full score is 20)

10. According to your knowledge, what are the transmission ways of COVID-19? [multiple choice] *

0= no, 1= yes (1 point) (Participants get 1 point for all correct answers)

□A. droplet transmission □B. contact transmission □C. mosquito transmission

□D. fecal mouth transmission □E. I don't know

The correct answers are A and B.

11. It is known to you that COVID-19 is susceptible to [single choice] * 0= no, 1= yes (1 point) (Participants get 1 point for all correct answers)

□A. children susceptible □B. young and middle-aged people susceptible

□C. middle-aged people susceptible □D. the elderly are susceptible

□E. the general population is susceptible □F. do not know

The correct answer is E.

12. What do you know about the common symptoms of COVID-19 infection? [multiple choice] *

0= no, 1= yes (1 point) (Participants get 1 point for all correct answers)

□A. dry cough □B. fever □C. shortness of breath, even difficulty

□D. fatigue □E. poor spirit, poor appetite □F. I don't know

The correct answers are A, B, C, D and E.

13. Which of the following do you think is an effective measure to prevent COVID-19 infection?

[multiple choice] * 0= no 1= yes (9 points) (Participants get 1 point for each right choice; Participants do not choose a wrong choice to get 1 point.)

□A. go out as little as possible, do not attend parties

□B. avoid contact with Wuhan returnees and fever

□C. wash hands with hand sanitizer, soap, etc.

□D. open Windows frequently for ventilation

□E. wear a mask when you go out

□F. Take virion, tamiflu, etc.

□G. Fumigation vinegar

□H. use medical alcohol to disinfect clothing, personal effects, etc.

□I. cover your mouth and nose with a tissue or elbow when sneezing or coughing

The correct answers are A, B, C, D, E, H and I.

14. Do you know how to wash your hands properly? [single choice] * (3 points) (A, B for 1 point; C for 2 points; D, E for 3 points)

□A. total ignorance □B. a little knowledge □C. general knowledge

□D. know a lot. □E. know a lot

15.Do you know the correct way to wear a mask? [single choice] * (3 points) (A, B for 1 point; C for 2 points; D, E for 3 points)

□A. total ignorance □B. a little knowledge □C. general knowledge

□D. know a lot. □E. know a lot

16. What do you think is true about COVID-19? [multiple choice] * (2 points) (Participants get 1 point for each right choice.)

□A. The incubation period is usually 3-7 days, with the longest not exceeding 14 days

□B. COVID-19 is also infectious during the incubation period

□C. I do not know.

The correct answers are A and B.

**Part four Risk Perception**

The following question is about your attitude towards COVID-19. There is no right or wrong answer. Please choose the most appropriate answer according to your own feelings. (Similar options in the attitude question are assigned to:1 point = impossible, 2 points = impossible, 3 points = impossible, 4 points = possible, 5 points = very possible, and the full score is 20.)

17. Even if a person is in good health, he may be infected with COVID-19*

□A. not at all □B. it won't happen. □C. it's hard to say

□D perhaps □E. very likely

18. I am more susceptible to infected COVID-19 than any other person*

□A. not at all □B. it won't happen □C. it's hard to say

□D. will appear □E. must be

19. Someone once reminded me to be careful of COVID-19*

□A. no one ever reminded me

□B. an occasional reminder

□C. it's hard to say

□D. they are often reminded

□E. be reminded all the time

20. I would be worried about my family being infected by COVID-19*

□A. not at all. □B. don't worry□ C. it's hard to say

□D. a little worried. □E. very worried

Thanks again for your support!
